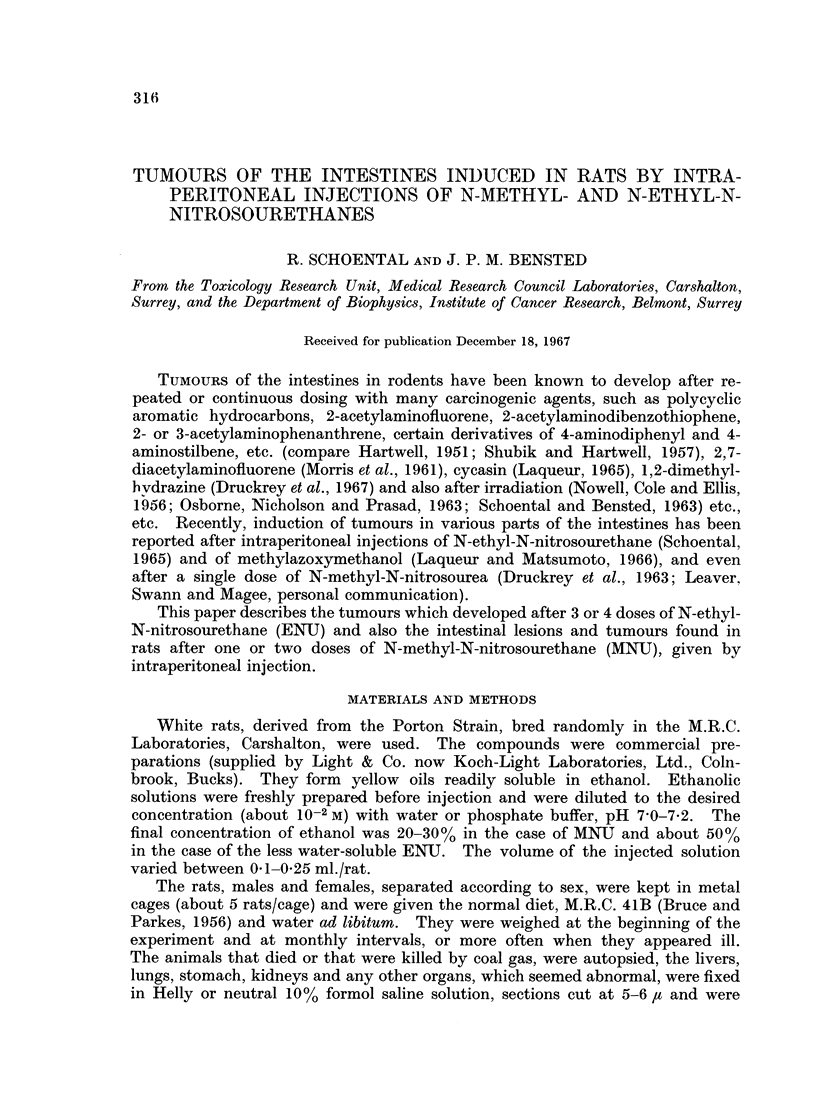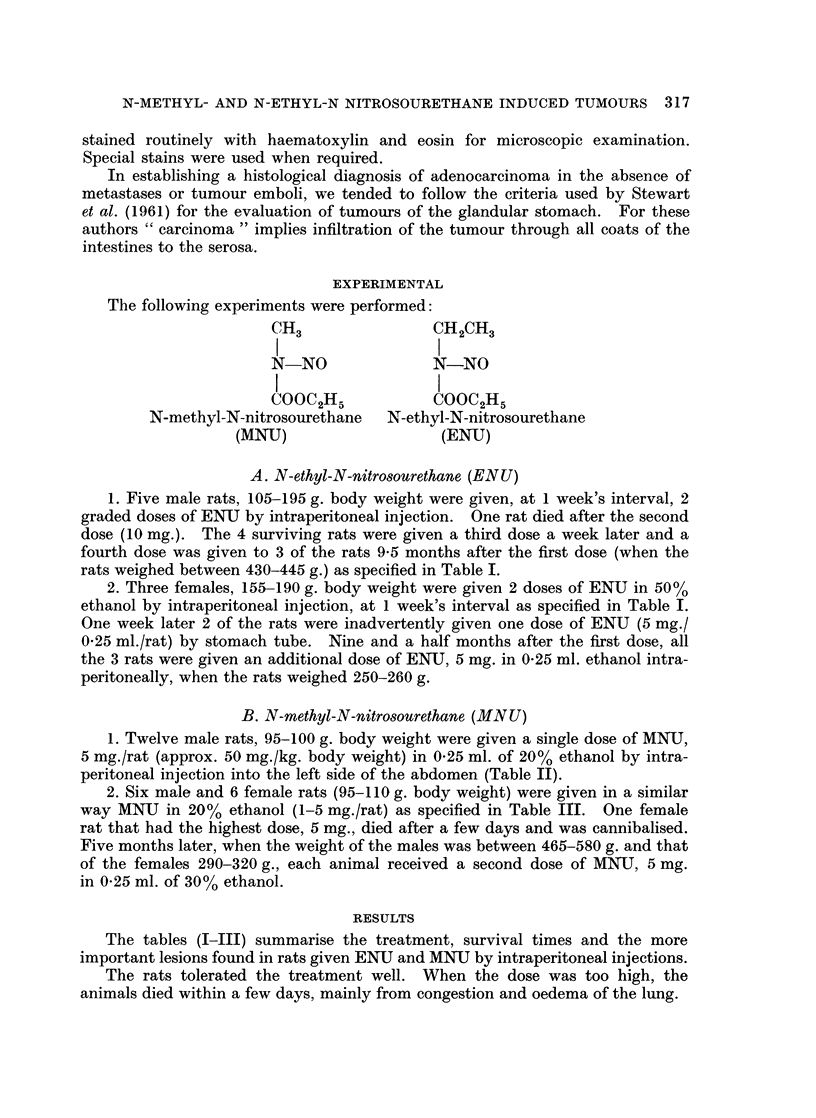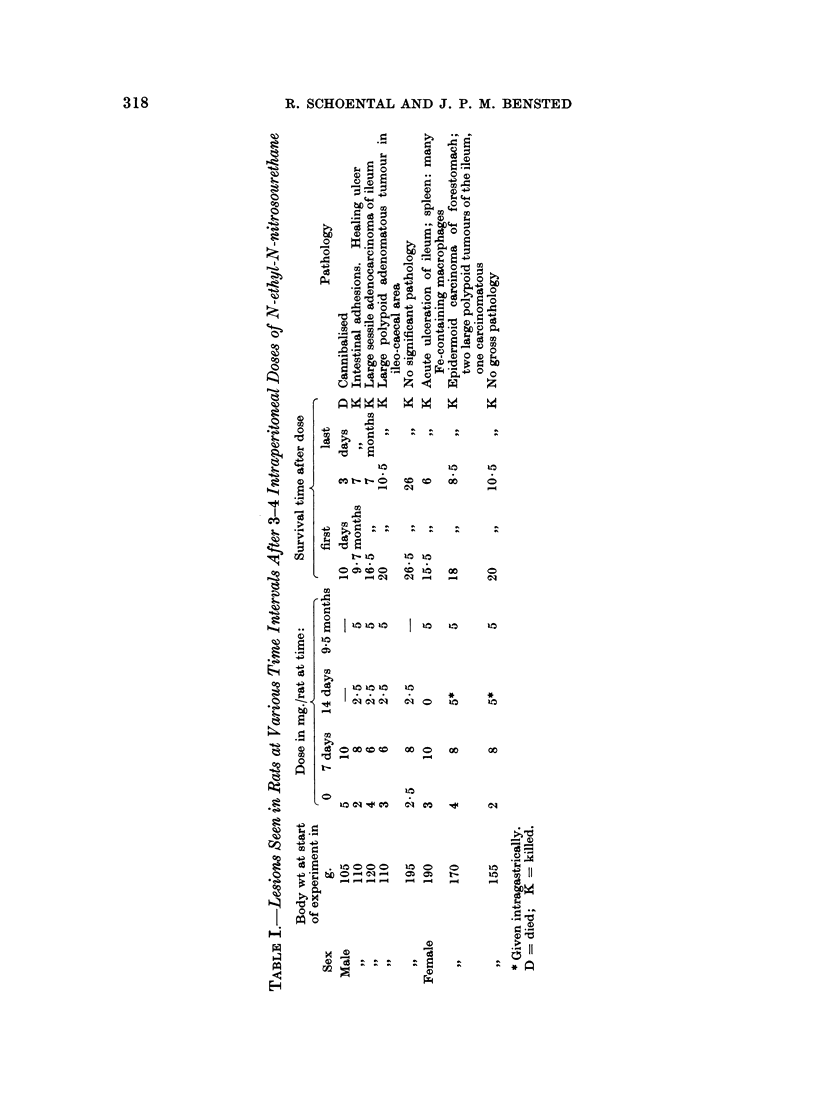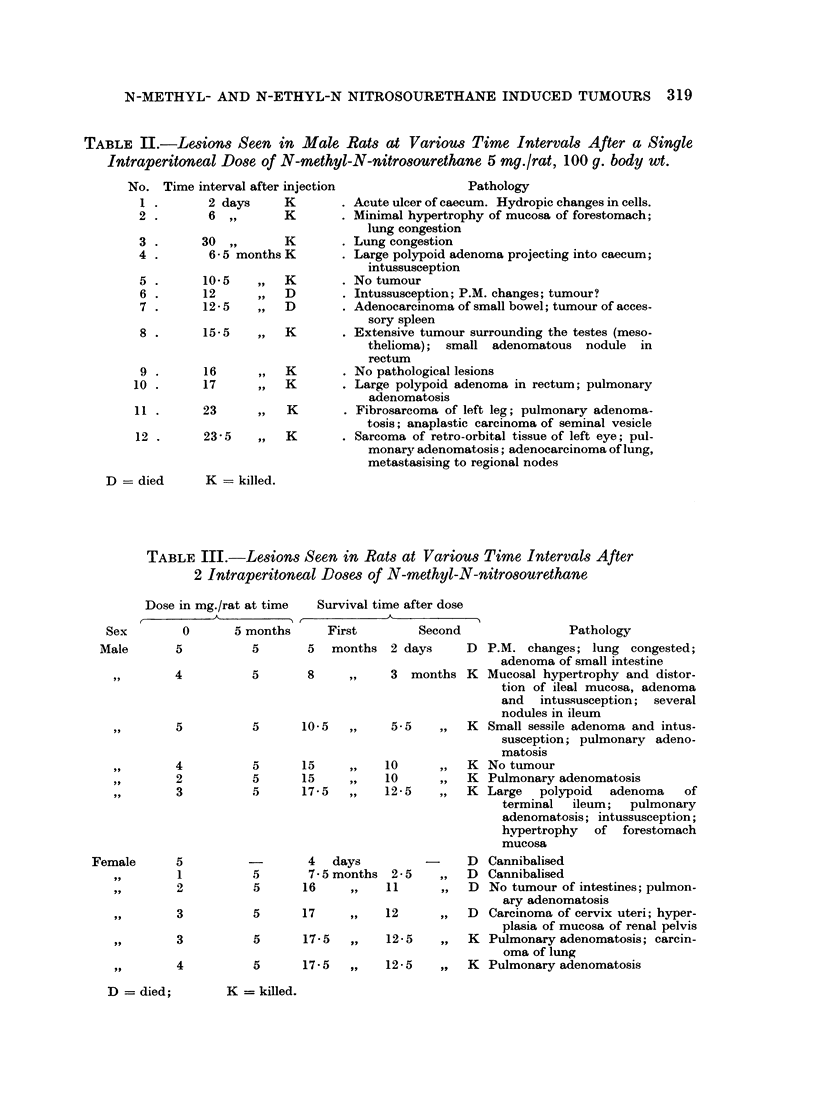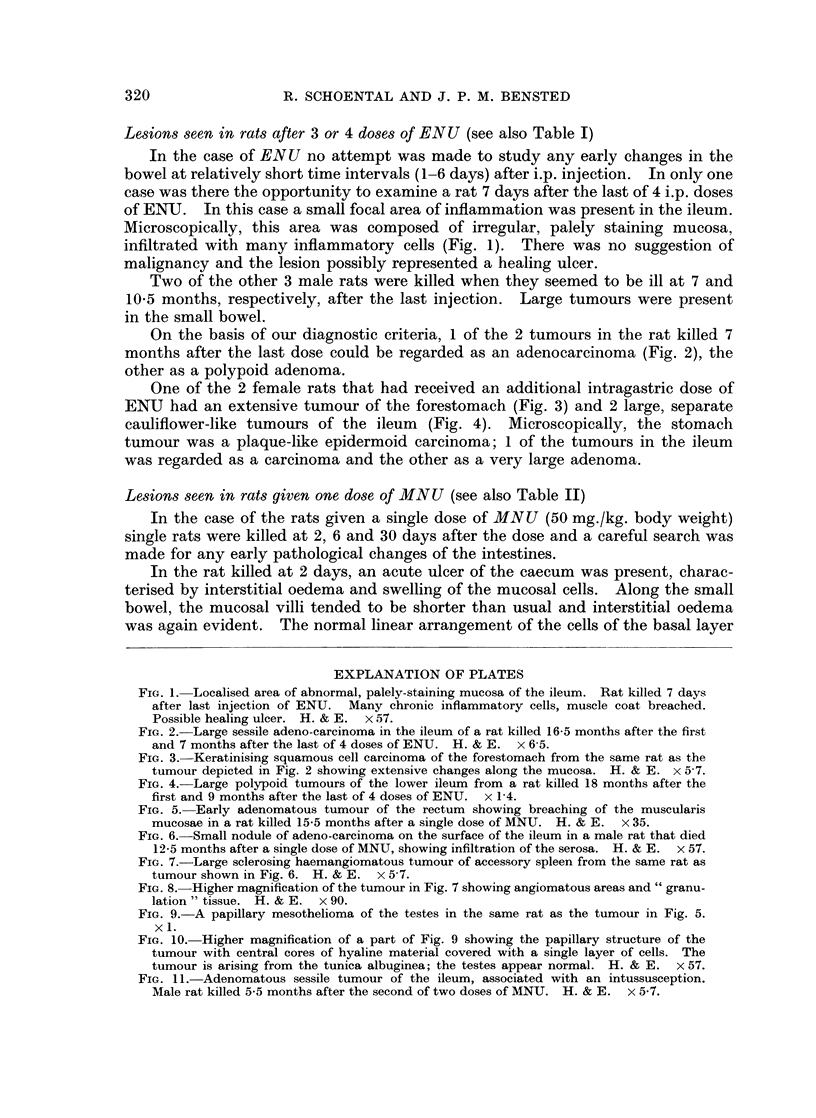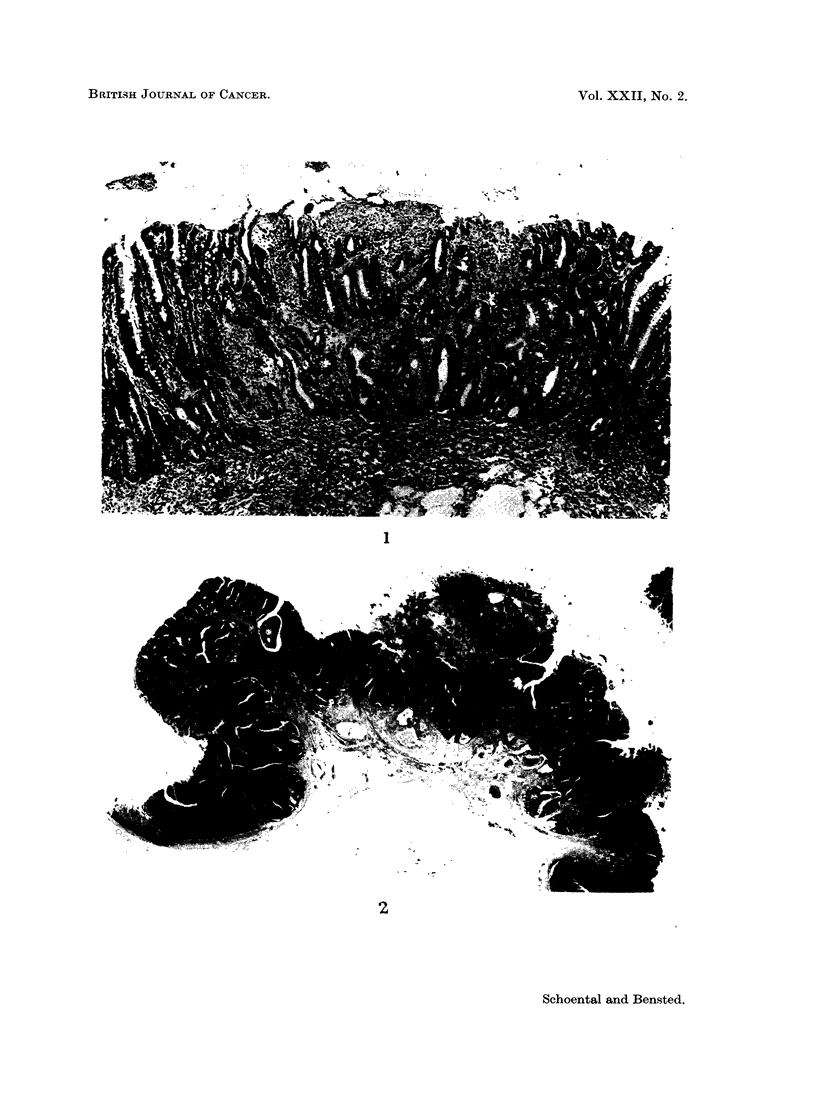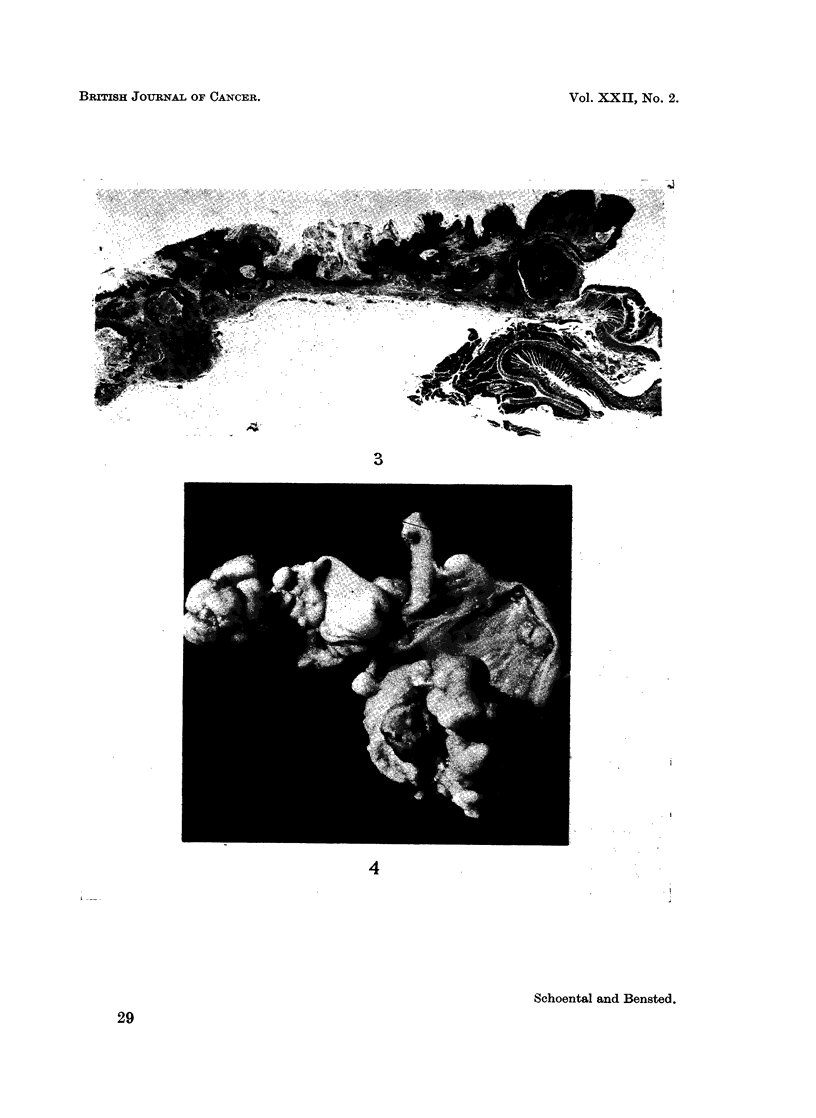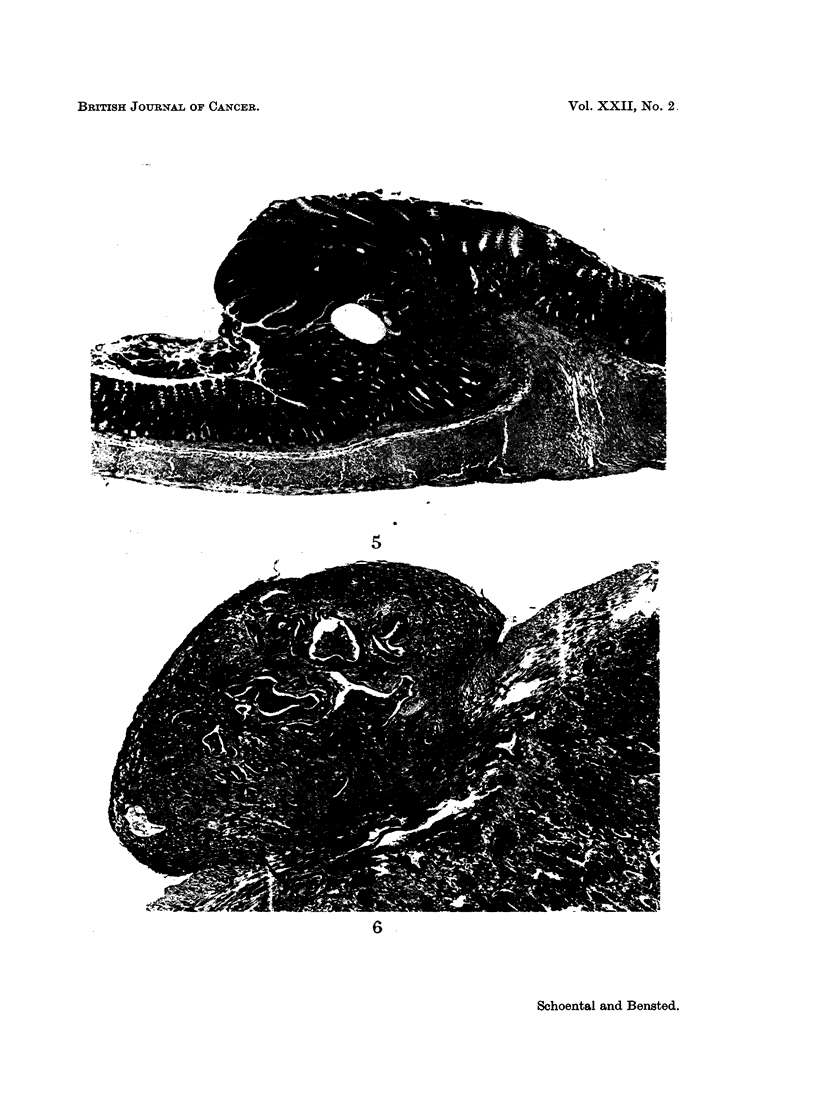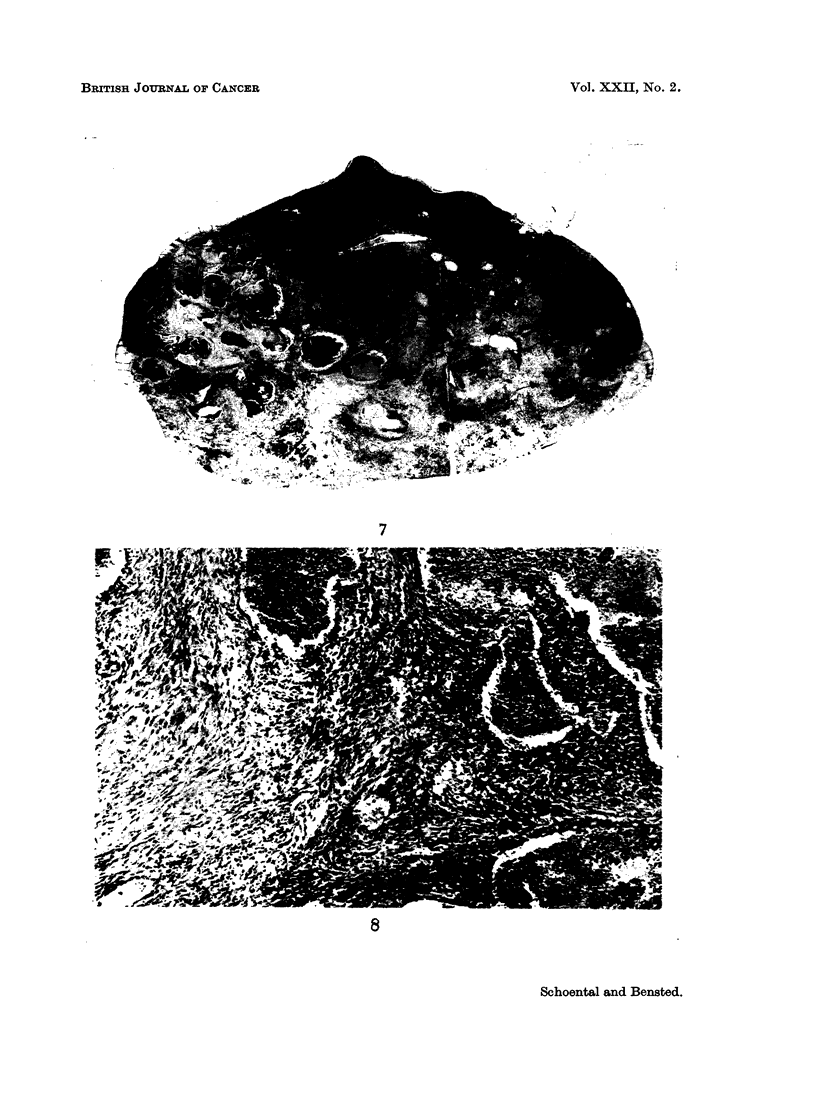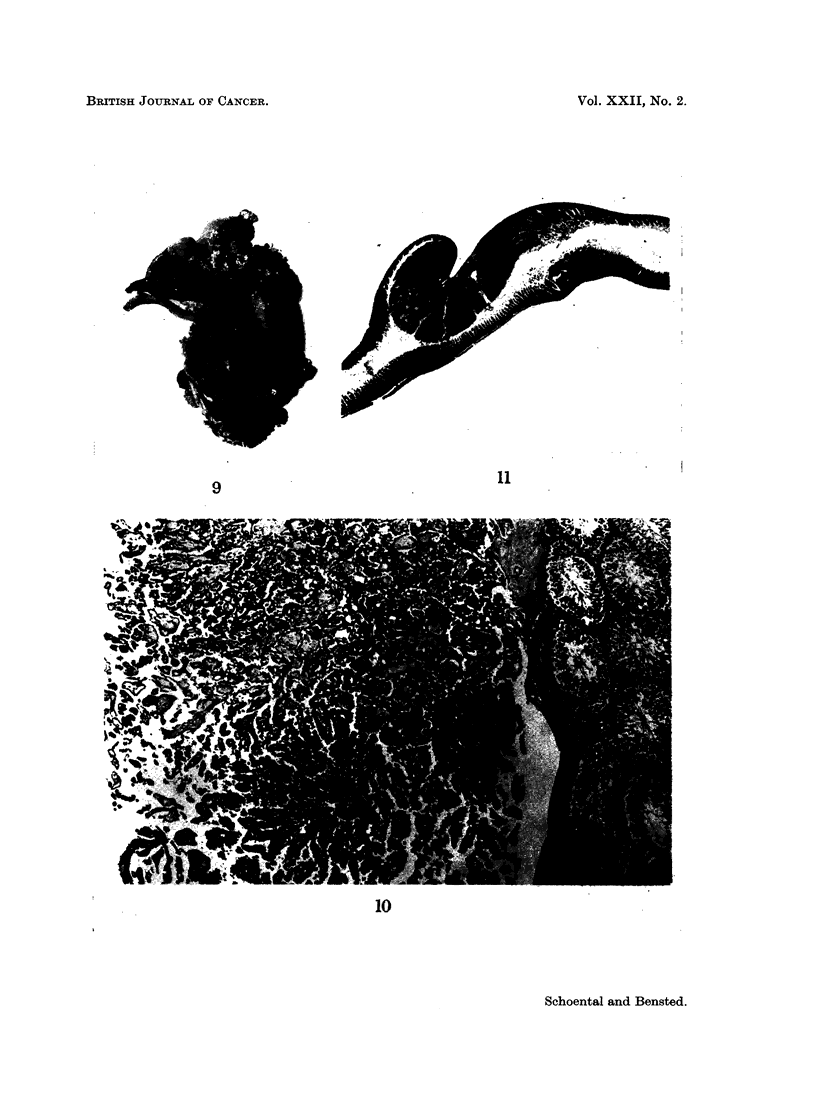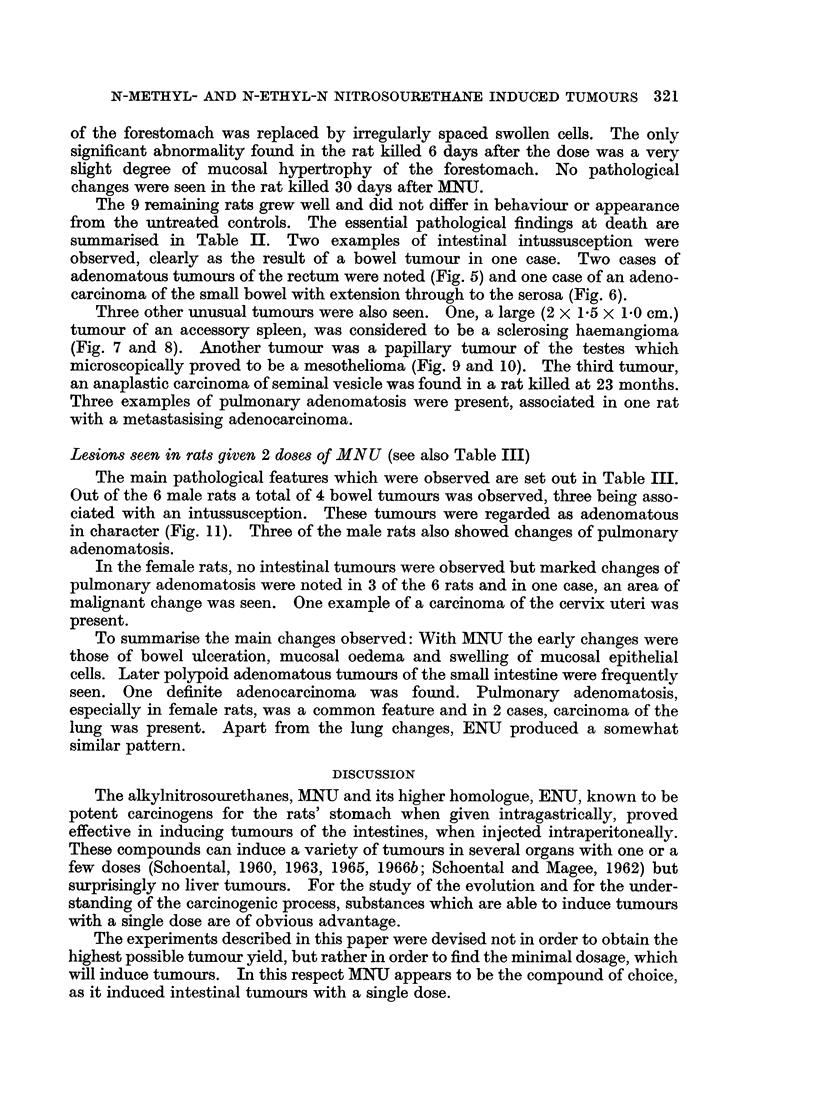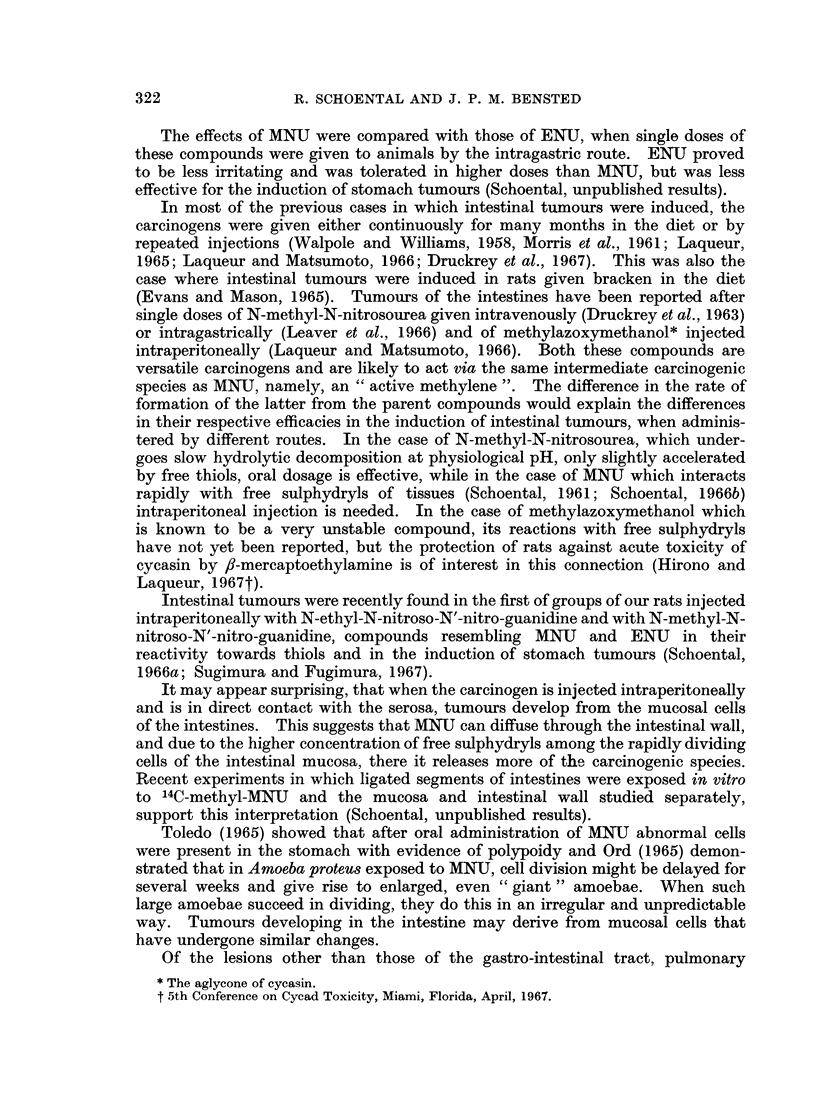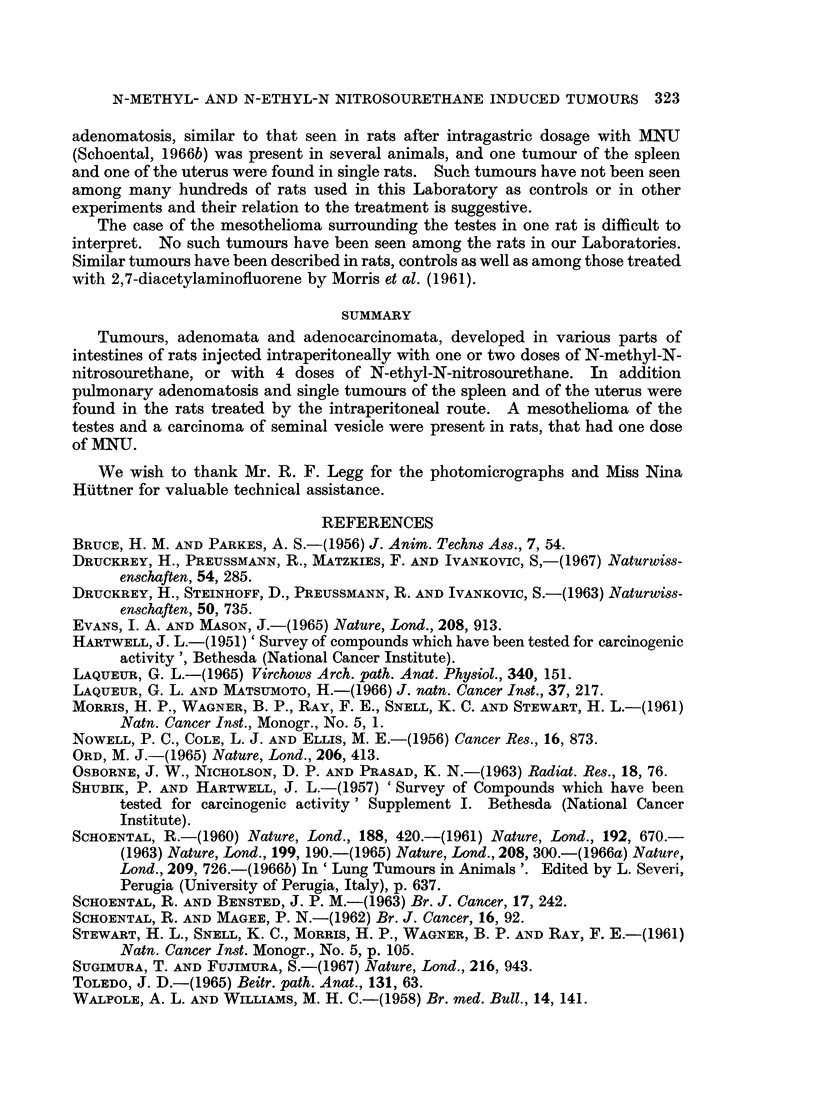# Tumours of the intestines induced in rats by intraperitoneal injections of N-methyl- and N-ethyl-N-nitrosourethanes.

**DOI:** 10.1038/bjc.1968.39

**Published:** 1968-06

**Authors:** R. Schoental, J. P. Bensted

## Abstract

**Images:**


### TUMOURS OF THE INTESTINES INDUCED IN RATS BY INTRA-PERITONEAL INJECTIONS OF N-METHYL- AND N-ETHYL-N-NITROSOURETHANES

R. SCHOENTAL AND J. P. M. BENSTED

*From the Toxicology Research Unit, Medical Research Council Laboratories, Carshalton, Surrey, and the Department of Biophysics, Institute of Cancer Research, Belmont, Surrey*

Received for publication December 18, 1967

TUMOURS of the intestines in rodents have been known to develop after repeated or continuous dosing with many carcinogenic agents, such as polycyclic aromatic hydrocarbons, 2-acetylaminofluorene, 2-acetylaminodibenzothiophene, 2- or 3-acetylaminophenanthrene, certain derivatives of 4-aminodiphenyl and 4-aminostilbene, etc. (compare Hartwell, 1951; Shubik and Hartwell, 1957), 2,7-diacetylaminofluorene (Morris *et al.*, 1961), cycasin (Laqueur, 1965), 1,2-dimethylhydrazine (Druckrey *et al.*, 1967) and also after irradiation (Nowell, Cole and Ellis, 1956; Osborne, Nicholson and Prasad, 1963; Schoental and Bensted, 1963) etc., etc. Recently, induction of tumours in various parts of the intestines has been reported after intraperitoneal injections of N-ethyl-N-nitrosourethane (Schoental, 1965) and of methylazoxymethanol (Laqueur and Matsumoto, 1966), and even after a single dose of N-methyl-N-nitrosourea (Druckrey *et al.*, 1963; Leaver, Swann and Magee, personal communication).

This paper describes the tumours which developed after 3 or 4 doses of N-ethyl-N-nitrosourethane (ENU) and also the intestinal lesions and tumours found in rats after one or two doses of N-methyl-N-nitrosourethane (MNU), given by intraperitoneal injection.

### MATERIALS AND METHODS

White rats, derived from the Porton Strain, bred randomly in the M.R.C. Laboratories, Carshalton, were used. The compounds were commercial preparations (supplied by Light & Co. now Koch-Light Laboratories, Ltd., Colnbrook, Bucks). They form yellow oils readily soluble in ethanol. Ethanolic solutions were freshly prepared before injection and were diluted to the desired concentration (about $10^{-2}$ M) with water or phosphate buffer, pH 7·0–7·2. The final concentration of ethanol was 20–30% in the case of MNU and about 50% in the case of the less water-soluble ENU. The volume of the injected solution varied between 0·1–0·25 ml./rat.

The rats, males and females, separated according to sex, were kept in metal cages (about 5 rats/cage) and were given the normal diet, M.R.C. 41B (Bruce and Parkes, 1956) and water *ad libitum*. They were weighed at the beginning of the experiment and at monthly intervals, or more often when they appeared ill. The animals that died or that were killed by coal gas, were autopsied, the livers, lungs, stomach, kidneys and any other organs, which seemed abnormal, were fixed in Helly or neutral 10% formol saline solution, sections cut at 5–6 $\mu$ and were stained routinely with haematoxylin and eosin for microscopic examination. Special stains were used when required.

In establishing a histological diagnosis of adenocarcinoma in the absence of metastases or tumour emboli, we tended to follow the criteria used by Stewart *et al.* (1961) for the evaluation of tumours of the glandular stomach. For these authors " carcinoma " implies infiltration of the tumour through all coats of the intestines to the serosa.

### EXPERIMENTAL

The following experiments were performed:

| | |
|---|---|
| $CH_3$ | $CH_2CH_3$ |
| \| | \| |
| N—NO | N—NO |
| \| | \| |
| $COOC_2H_5$ | $COOC_2H_5$ |
| N-methyl-N-nitrosourethane (MNU) | N-ethyl-N-nitrosourethane (ENU) |

### *A. N-ethyl-N-nitrosourethane (ENU)*

1. Five male rats, 105–195 g. body weight were given, at 1 week's interval, 2 graded doses of ENU by intraperitoneal injection. One rat died after the second dose (10 mg.). The 4 surviving rats were given a third dose a week later and a fourth dose was given to 3 of the rats 9·5 months after the first dose (when the rats weighed between 430–445 g.) as specified in Table I.

2. Three females, 155–190 g. body weight were given 2 doses of ENU in 50% ethanol by intraperitoneal injection, at 1 week's interval as specified in Table I. One week later 2 of the rats were inadvertently given one dose of ENU (5 mg./0·25 ml./rat) by stomach tube. Nine and a half months after the first dose, all the 3 rats were given an additional dose of ENU, 5 mg. in 0·25 ml. ethanol intraperitoneally, when the rats weighed 250–260 g.

### *B. N-methyl-N-nitrosourethane (MNU)*

1. Twelve male rats, 95–100 g. body weight were given a single dose of MNU, 5 mg./rat (approx. 50 mg./kg. body weight) in 0·25 ml. of 20% ethanol by intraperitoneal injection into the left side of the abdomen (Table II).

2. Six male and 6 female rats (95–110 g. body weight) were given in a similar way MNU in 20% ethanol (1–5 mg./rat) as specified in Table III. One female rat that had the highest dose, 5 mg., died after a few days and was cannibalised. Five months later, when the weight of the males was between 465–580 g. and that of the females 290–320 g., each animal received a second dose of MNU, 5 mg. in 0·25 ml. of 30% ethanol.

### RESULTS

The tables (I–III) summarise the treatment, survival times and the more important lesions found in rats given ENU and MNU by intraperitoneal injections.

The rats tolerated the treatment well. When the dose was too high, the animals died within a few days, mainly from congestion and oedema of the lung.

TABLE I.—*Lesions Seen in Rats at Various Time Intervals After 3–4 Intraperitoneal Doses of N-ethyl-N-nitrosourethane*

| Sex | Body wt at start of experiment in g. | Dose in mg./rat at time: 0 | 7 days | 14 days | 9·5 months | Survival time after dose: first | last | | Pathology |
|---|---|---|---|---|---|---|---|---|---|
| Male | 105 | 5 | 10 | — | — | 10 days | 3 days | D | Cannibalised |
| ,, | 110 | 2 | 8 | 2·5 | 5 | 9·7 months | 7 ,, | K | Intestinal adhesions. Healing ulcer |
| ,, | 120 | 4 | 6 | 2·5 | 5 | 16·5 ,, | 7 months | K | Large sessile adenocarcinoma of ileum |
| ,, | 110 | 3 | 6 | 2·5 | 5 | 20 ,, | 10·5 ,, | K | Large polypoid adenomatous tumour in ileo-caecal area |
| ,, | 195 | 2·5 | 8 | 2·5 | — | 26·5 ,, | 26 ,, | K | No significant pathology |
| Female | 190 | 3 | 10 | 0 | 5 | 15·5 ,, | 6 ,, | K | Acute ulceration of ileum; spleen: many Fe-containing macrophages |
| ,, | 170 | 4 | 8 | 5* | 5 | 18 ,, | 8·5 ,, | K | Epidermoid carcinoma of forestomach; two large polypoid tumours of the ileum, one carcinomatous |
| ,, | 155 | 2 | 8 | 5* | 5 | 20 ,, | 10·5 ,, | K | No gross pathology |

* Given intragastrically.

D = died; K = killed.

TABLE II.—*Lesions Seen in Male Rats at Various Time Intervals After a Single Intraperitoneal Dose of N-methyl-N-nitrosourethane 5 mg./rat, 100 g. body wt.*

| No. | Time interval after injection | | Pathology |
|---|---|---|---|
| 1 | 2 days | K | Acute ulcer of caecum. Hydropic changes in cells. |
| 2 | 6 „ | K | Minimal hypertrophy of mucosa of forestomach; lung congestion |
| 3 | 30 „ | K | Lung congestion |
| 4 | 6·5 months | K | Large polypoid adenoma projecting into caecum; intussusception |
| 5 | 10·5 „ | K | No tumour |
| 6 | 12 „ | D | Intussusception; P.M. changes; tumour? |
| 7 | 12·5 „ | D | Adenocarcinoma of small bowel; tumour of accessory spleen |
| 8 | 15·5 „ | K | Extensive tumour surrounding the testes (mesothelioma); small adenomatous nodule in rectum |
| 9 | 16 „ | K | No pathological lesions |
| 10 | 17 „ | K | Large polypoid adenoma in rectum; pulmonary adenomatosis |
| 11 | 23 „ | K | Fibrosarcoma of left leg; pulmonary adenomatosis; anaplastic carcinoma of seminal vesicle |
| 12 | 23·5 „ | K | Sarcoma of retro-orbital tissue of left eye; pulmonary adenomatosis; adenocarcinoma of lung, metastasising to regional nodes |

D = died K = killed.

TABLE III.—*Lesions Seen in Rats at Various Time Intervals After 2 Intraperitoneal Doses of N-methyl-N-nitrosourethane*

| | Dose in mg./rat at time | | Survival time after dose | | | |
|---|---|---|---|---|---|---|
| Sex | 0 | 5 months | First | Second | | Pathology |
| Male | 5 | 5 | 5 months | 2 days | D | P.M. changes; lung congested; adenoma of small intestine |
| „ | 4 | 5 | 8 „ | 3 months | K | Mucosal hypertrophy and distortion of ileal mucosa, adenoma and intussusception; several nodules in ileum |
| „ | 5 | 5 | 10·5 „ | 5·5 „ | K | Small sessile adenoma and intussusception; pulmonary adenomatosis |
| „ | 4 | 5 | 15 „ | 10 „ | K | No tumour |
| „ | 2 | 5 | 15 „ | 10 „ | K | Pulmonary adenomatosis |
| „ | 3 | 5 | 17·5 „ | 12·5 „ | K | Large polypoid adenoma of terminal ileum; pulmonary adenomatosis; intussusception; hypertrophy of forestomach mucosa |
| Female | 5 | — | 4 days | — | D | Cannibalised |
| „ | 1 | 5 | 7·5 months | 2·5 „ | D | Cannibalised |
| „ | 2 | 5 | 16 „ | 11 „ | D | No tumour of intestines; pulmonary adenomatosis |
| „ | 3 | 5 | 17 „ | 12 „ | D | Carcinoma of cervix uteri; hyperplasia of mucosa of renal pelvis |
| „ | 3 | 5 | 17·5 „ | 12·5 „ | K | Pulmonary adenomatosis; carcinoma of lung |
| „ | 4 | 5 | 17·5 „ | 12·5 „ | K | Pulmonary adenomatosis |

D = died; K = killed.

*Lesions seen in rats after 3 or 4 doses of ENU* (see also Table I)

In the case of *ENU* no attempt was made to study any early changes in the bowel at relatively short time intervals (1–6 days) after i.p. injection. In only one case was there the opportunity to examine a rat 7 days after the last of 4 i.p. doses of ENU. In this case a small focal area of inflammation was present in the ileum. Microscopically, this area was composed of irregular, palely staining mucosa, infiltrated with many inflammatory cells (Fig. 1). There was no suggestion of malignancy and the lesion possibly represented a healing ulcer.

Two of the other 3 male rats were killed when they seemed to be ill at 7 and 10·5 months, respectively, after the last injection. Large tumours were present in the small bowel.

On the basis of our diagnostic criteria, 1 of the 2 tumours in the rat killed 7 months after the last dose could be regarded as an adenocarcinoma (Fig. 2), the other as a polypoid adenoma.

One of the 2 female rats that had received an additional intragastric dose of ENU had an extensive tumour of the forestomach (Fig. 3) and 2 large, separate cauliflower-like tumours of the ileum (Fig. 4). Microscopically, the stomach tumour was a plaque-like epidermoid carcinoma; 1 of the tumours in the ileum was regarded as a carcinoma and the other as a very large adenoma.

*Lesions seen in rats given one dose of MNU* (see also Table II)

In the case of the rats given a single dose of *MNU* (50 mg./kg. body weight) single rats were killed at 2, 6 and 30 days after the dose and a careful search was made for any early pathological changes of the intestines.

In the rat killed at 2 days, an acute ulcer of the caecum was present, characterised by interstitial oedema and swelling of the mucosal cells. Along the small bowel, the mucosal villi tended to be shorter than usual and interstitial oedema was again evident. The normal linear arrangement of the cells of the basal layer

---

EXPLANATION OF PLATES

FIG. 1.—Localised area of abnormal, palely-staining mucosa of the ileum. Rat killed 7 days after last injection of ENU. Many chronic inflammatory cells, muscle coat breached. Possible healing ulcer. H. & E. ×57.

FIG. 2.—Large sessile adeno-carcinoma in the ileum of a rat killed 16·5 months after the first and 7 months after the last of 4 doses of ENU. H. & E. ×6·5.

FIG. 3.—Keratinising squamous cell carcinoma of the forestomach from the same rat as the tumour depicted in Fig. 2 showing extensive changes along the mucosa. H. & E. ×5·7.

FIG. 4.—Large polypoid tumours of the lower ileum from a rat killed 18 months after the first and 9 months after the last of 4 doses of ENU. ×1·4.

FIG. 5.—Early adenomatous tumour of the rectum showing breaching of the muscularis mucosae in a rat killed 15·5 months after a single dose of MNU. H. & E. ×35.

FIG. 6.—Small nodule of adeno-carcinoma on the surface of the ileum in a male rat that died 12·5 months after a single dose of MNU, showing infiltration of the serosa. H. & E. ×57.

FIG. 7.—Large sclerosing haemangiomatous tumour of accessory spleen from the same rat as tumour shown in Fig. 6. H. & E. ×5·7.

FIG. 8.—Higher magnification of the tumour in Fig. 7 showing angiomatous areas and "granulation" tissue. H. & E. ×90.

FIG. 9.—A papillary mesothelioma of the testes in the same rat as the tumour in Fig. 5. ×1.

FIG. 10.—Higher magnification of a part of Fig. 9 showing the papillary structure of the tumour with central cores of hyaline material covered with a single layer of cells. The tumour is arising from the tunica albuginea; the testes appear normal. H. & E. ×57.

FIG. 11.—Adenomatous sessile tumour of the ileum, associated with an intussusception. Male rat killed 5·5 months after the second of two doses of MNU. H. & E. ×5·7.

1

2

Schoental and Bensted.

3

4

5

6

Schoental and Bensted.

7

8

Schoental and Bensted.

9

11

10

of the forestomach was replaced by irregularly spaced swollen cells. The only significant abnormality found in the rat killed 6 days after the dose was a very slight degree of mucosal hypertrophy of the forestomach. No pathological changes were seen in the rat killed 30 days after MNU.

The 9 remaining rats grew well and did not differ in behaviour or appearance from the untreated controls. The essential pathological findings at death are summarised in Table II. Two examples of intestinal intussusception were observed, clearly as the result of a bowel tumour in one case. Two cases of adenomatous tumours of the rectum were noted (Fig. 5) and one case of an adenocarcinoma of the small bowel with extension through to the serosa (Fig. 6).

Three other unusual tumours were also seen. One, a large (2 × 1·5 × 1·0 cm.) tumour of an accessory spleen, was considered to be a sclerosing haemangioma (Fig. 7 and 8). Another tumour was a papillary tumour of the testes which microscopically proved to be a mesothelioma (Fig. 9 and 10). The third tumour, an anaplastic carcinoma of seminal vesicle was found in a rat killed at 23 months. Three examples of pulmonary adenomatosis were present, associated in one rat with a metastasising adenocarcinoma.

*Lesions seen in rats given 2 doses of MNU* (see also Table III)

The main pathological features which were observed are set out in Table III. Out of the 6 male rats a total of 4 bowel tumours was observed, three being associated with an intussusception. These tumours were regarded as adenomatous in character (Fig. 11). Three of the male rats also showed changes of pulmonary adenomatosis.

In the female rats, no intestinal tumours were observed but marked changes of pulmonary adenomatosis were noted in 3 of the 6 rats and in one case, an area of malignant change was seen. One example of a carcinoma of the cervix uteri was present.

To summarise the main changes observed: With MNU the early changes were those of bowel ulceration, mucosal oedema and swelling of mucosal epithelial cells. Later polypoid adenomatous tumours of the small intestine were frequently seen. One definite adenocarcinoma was found. Pulmonary adenomatosis, especially in female rats, was a common feature and in 2 cases, carcinoma of the lung was present. Apart from the lung changes, ENU produced a somewhat similar pattern.

### DISCUSSION

The alkylnitrosourethanes, MNU and its higher homologue, ENU, known to be potent carcinogens for the rats' stomach when given intragastrically, proved effective in inducing tumours of the intestines, when injected intraperitoneally. These compounds can induce a variety of tumours in several organs with one or a few doses (Schoental, 1960, 1963, 1965, 1966*b*; Schoental and Magee, 1962) but surprisingly no liver tumours. For the study of the evolution and for the understanding of the carcinogenic process, substances which are able to induce tumours with a single dose are of obvious advantage.

The experiments described in this paper were devised not in order to obtain the highest possible tumour yield, but rather in order to find the minimal dosage, which will induce tumours. In this respect MNU appears to be the compound of choice, as it induced intestinal tumours with a single dose.

The effects of MNU were compared with those of ENU, when single doses of these compounds were given to animals by the intragastric route. ENU proved to be less irritating and was tolerated in higher doses than MNU, but was less effective for the induction of stomach tumours (Schoental, unpublished results).

In most of the previous cases in which intestinal tumours were induced, the carcinogens were given either continuously for many months in the diet or by repeated injections (Walpole and Williams, 1958, Morris *et al.*, 1961; Laqueur, 1965; Laqueur and Matsumoto, 1966; Druckrey *et al.*, 1967). This was also the case where intestinal tumours were induced in rats given bracken in the diet (Evans and Mason, 1965). Tumours of the intestines have been reported after single doses of N-methyl-N-nitrosourea given intravenously (Druckrey *et al.*, 1963) or intragastrically (Leaver *et al.*, 1966) and of methylazoxymethanol* injected intraperitoneally (Laqueur and Matsumoto, 1966). Both these compounds are versatile carcinogens and are likely to act *via* the same intermediate carcinogenic species as MNU, namely, an " active methylene ". The difference in the rate of formation of the latter from the parent compounds would explain the differences in their respective efficacies in the induction of intestinal tumours, when administered by different routes. In the case of N-methyl-N-nitrosourea, which undergoes slow hydrolytic decomposition at physiological pH, only slightly accelerated by free thiols, oral dosage is effective, while in the case of MNU which interacts rapidly with free sulphydryls of tissues (Schoental, 1961; Schoental, 1966*b*) intraperitoneal injection is needed. In the case of methylazoxymethanol which is known to be a very unstable compound, its reactions with free sulphydryls have not yet been reported, but the protection of rats against acute toxicity of cycasin by $\beta$-mercaptoethylamine is of interest in this connection (Hirono and Laqueur, 1967†).

Intestinal tumours were recently found in the first of groups of our rats injected intraperitoneally with N-ethyl-N-nitroso-N′-nitro-guanidine and with N-methyl-N-nitroso-N′-nitro-guanidine, compounds resembling MNU and ENU in their reactivity towards thiols and in the induction of stomach tumours (Schoental, 1966*a*; Sugimura and Fugimura, 1967).

It may appear surprising, that when the carcinogen is injected intraperitoneally and is in direct contact with the serosa, tumours develop from the mucosal cells of the intestines. This suggests that MNU can diffuse through the intestinal wall, and due to the higher concentration of free sulphydryls among the rapidly dividing cells of the intestinal mucosa, there it releases more of the carcinogenic species. Recent experiments in which ligated segments of intestines were exposed *in vitro* to $^{14}C$-methyl-MNU and the mucosa and intestinal wall studied separately, support this interpretation (Schoental, unpublished results).

Toledo (1965) showed that after oral administration of MNU abnormal cells were present in the stomach with evidence of polypoidy and Ord (1965) demonstrated that in *Amoeba proteus* exposed to MNU, cell division might be delayed for several weeks and give rise to enlarged, even " giant " amoebae. When such large amoebae succeed in dividing, they do this in an irregular and unpredictable way. Tumours developing in the intestine may derive from mucosal cells that have undergone similar changes.

Of the lesions other than those of the gastro-intestinal tract, pulmonary

* The aglycone of cycasin.

† 5th Conference on Cycad Toxicity, Miami, Florida, April, 1967.

adenomatosis, similar to that seen in rats after intragastric dosage with MNU (Schoental, 1966*b*) was present in several animals, and one tumour of the spleen and one of the uterus were found in single rats. Such tumours have not been seen among many hundreds of rats used in this Laboratory as controls or in other experiments and their relation to the treatment is suggestive.

The case of the mesothelioma surrounding the testes in one rat is difficult to interpret. No such tumours have been seen among the rats in our Laboratories. Similar tumours have been described in rats, controls as well as among those treated with 2,7-diacetylaminofluorene by Morris *et al.* (1961).

### SUMMARY

Tumours, adenomata and adenocarcinomata, developed in various parts of intestines of rats injected intraperitoneally with one or two doses of N-methyl-N-nitrosourethane, or with 4 doses of N-ethyl-N-nitrosourethane. In addition pulmonary adenomatosis and single tumours of the spleen and of the uterus were found in the rats treated by the intraperitoneal route. A mesothelioma of the testes and a carcinoma of seminal vesicle were present in rats, that had one dose of MNU.

We wish to thank Mr. R. F. Legg for the photomicrographs and Miss Nina Hüttner for valuable technical assistance.

### REFERENCES

BRUCE, H. M. AND PARKES, A. S.—(1956) *J. Anim. Techns Ass.*, **7**, 54.

DRUCKREY, H., PREUSSMANN, R., MATZKIES, F. AND IVANKOVIC, S,—(1967) *Naturwissenschaften*, **54**, 285.

DRUCKREY, H., STEINHOFF, D., PREUSSMANN, R. AND IVANKOVIC, S.—(1963) *Naturwissenschaften*, **50**, 735.

EVANS, I. A. AND MASON, J.—(1965) *Nature, Lond.*, **208**, 913.

HARTWELL, J. L.—(1951) ' Survey of compounds which have been tested for carcinogenic activity ', Bethesda (National Cancer Institute).

LAQUEUR, G. L.—(1965) *Virchows Arch. path. Anat. Physiol.*, **340**, 151.

LAQUEUR, G. L. AND MATSUMOTO, H.—(1966) *J. natn. Cancer Inst.*, **37**, 217.

MORRIS, H. P., WAGNER, B. P., RAY, F. E., SNELL, K. C. AND STEWART, H. L.—(1961) *Natn. Cancer Inst.*, Monogr., No. 5, 1.

NOWELL, P. C., COLE, L. J. AND ELLIS, M. E.—(1956) *Cancer Res.*, **16**, 873.

ORD, M. J.—(1965) *Nature, Lond.*, **206**, 413.

OSBORNE, J. W., NICHOLSON, D. P. AND PRASAD, K. N.—(1963) *Radiat. Res.*, **18**, 76.

SHUBIK, P. AND HARTWELL, J. L.—(1957) ' Survey of Compounds which have been tested for carcinogenic activity ' Supplement I. Bethesda (National Cancer Institute).

SCHOENTAL, R.—(1960) *Nature, Lond.*, **188**, 420.—(1961) *Nature, Lond.*, **192**, 670.—(1963) *Nature, Lond.*, **199**, 190.—(1965) *Nature, Lond.*, **208**, 300.—(1966*a*) *Nature, Lond.*, **209**, 726.—(1966*b*) In ' Lung Tumours in Animals '. Edited by L. Severi, Perugia (University of Perugia, Italy), p. 637.

SCHOENTAL, R. AND BENSTED, J. P. M.—(1963) *Br. J. Cancer*, **17**, 242.

SCHOENTAL, R. AND MAGEE, P. N.—(1962) *Br. J. Cancer*, **16**, 92.

STEWART, H. L., SNELL, K. C., MORRIS, H. P., WAGNER, B. P. AND RAY, F. E.—(1961) *Natn. Cancer Inst.* Monogr., No. 5, p. 105.

SUGIMURA, T. AND FUJIMURA, S.—(1967) *Nature, Lond.*, **216**, 943.

TOLEDO, J. D.—(1965) *Beitr. path. Anat.*, **131**, 63.

WALPOLE, A. L. AND WILLIAMS, M. H. C.—(1958) *Br. med. Bull.*, **14**, 141.